# Spatial Distribution of Glycerophospholipids in the Ocular Lens

**DOI:** 10.1371/journal.pone.0019441

**Published:** 2011-04-29

**Authors:** Jaroslav Pól, Veronika Vidová, Tuulia Hyötyläinen, Michael Volný, Petr Novák, Martin Strohalm, Risto Kostiainen, Vladimír Havlíček, Susanne K. Wiedmer, Juha M. Holopainen

**Affiliations:** 1 Laboratory of Molecular Structure Characterization, Institute of Microbiology, Academy of Sciences of the Czech Republic, Prague, Czech Republic; 2 Division of Pharmaceutical Chemistry, Faculty of Pharmacy, University of Helsinki, Helsinki, Finland; 3 Department of Analytical Chemistry, Faculty of Science, Palacký University, Olomouc, Czech Republic; 4 VTT Technical Research Centre of Finland, Espoo, Finland; 5 Laboratory of Analytical Chemistry, Department of Chemistry, University of Helsinki, Helsinki, Finland; 6 Helsinki Eye Lab, Department of Ophthalmology, University of Helsinki, Helsinki, Finland; Université Joseph Fourier, France

## Abstract

Knowledge of the spatial distribution of lipids in the intraocular lens is important for understanding the physiology and biochemistry of this unique tissue and for gaining a better insight into the mechanisms underlying diseases of the lens. Following our previous study showing the spatial distribution of sphingolipids in the porcine lens, the current study used ultra performance liquid chromatography quadrupole time-of-flight mass spectrometry (UPLC-QTOFMS) to provide the whole lipidome of porcine lens and these studies were supplemented by matrix-assisted laser desorption ionization mass spectrometry imaging (MALDI MSI) of the lens using ultra-high resolution Fourier transform ion cyclotron resonance mass spectrometry (FTICR MS) to determine the spatial distribution of glycerophospholipids. Altogether 172 lipid species were identified with high confidence and their concentration was determined. Sphingomyelins, phosphatidylcholines, and phosphatidylethanolamines were the most abundant lipid classes. We then determined the spatial and concentration-dependent distributions of 20 phosphatidylcholines, 6 phosphatidylethanolamines, and 4 phosphatidic acids. Based on the planar molecular images of the lipids, we report the organization of fiber cell membranes within the ocular lens and suggest roles for these lipids in normal and diseased lenses.

## Introduction

The ocular lens is a unique epithelial tissue responsible for fine-tuning images projected onto the retina. To present a sharp and clear image, the lens needs to be transparent, minimize light scattering, and must be able to change its curvature. The mammalian lens is composed of lens fiber cells derived from a single layer of lenticular epithelial cells. As the lens ages, the fiber cells elongate and the older cells are packed toward the center of the lens, forming concentric onion-like layers of fiber cells. The oldest cells are positioned in the innermost segment of the lens (nucleus), whereas the younger cells are located in the cortical region (cortex) of the lens. The lens fiber cells are precisely aligned with the adjacent cells and also contain high amounts of crystallins, specialized soluble proteins. These pzroteins give the fiber cells a significantly higher refractive index than the fluids around the lens and also reduce light scattering [1]. Due to the loss of internal organelles from the fiber cells [2], the plasma membrane eventually becomes the only cellular organ of the lens. This membrane is unique in that it contains high concentrations of the integral membrane protein aquaporin 0 (AQP0), a 26 kDa water channel, and in that it lacks polyunsaturated phospholipids and contains high concentrations of sphingolipids and cholesterol [3]–[9].

Mass spectrometry imaging (MSI) allows for the visualization of molecular maps through its automated raster-collection of mass spectra from a tissue surface [10]. Several direct desorption ionization techniques have been developed to operate in MSI mode. Matrix-assisted laser desorption ionization (MALDI) [11], [12], desorption electrospray ionization (DESI) [13], and secondary ion mass spectrometry (SIMS) [14] are the most common techniques. As an emerging technology, MSI has already been targeted to address biological (mal)functions [15], [16], and this technique and its applications have been reviewed [17], [18] and a protocol has been established [19]. MSI benefits from its power to visualize spatial chemical distribution across a tissue. One of the limitations of MSI is problematic quantification. Desorption and ionization of a compound from a tissue during MALDI MSI is influenced by the MALDI matrix type, its thickness, size of crystals, and the quality of the tissue surface. Diverse anatomy of a tissue (accompanied by native chemical changes) is responsible for variations of MALDI signal response. The use of internal standard in MALDI MSI has not yet been established and therefore only relative quantitative changes of an ion signal can be measured. Accordingly, absolute concentrations cannot be obtained and thus it would be advisable to use other techniques allowing for quantitative assessment of the whole lipidome. Ocular lens is a tissue, which anatomy is simple and its nucleus and cortex forms quite uniform structure. Therefore we anticipate small variation in MALDI signal response across the tissue slice. Quantification of about 100 phospholipids in the whole human and animal intraocular lenses has been performed using ESI-MS [20]. That study showed phosphatidylcholines (PCs) to be the most abundant lipids in porcine lens, followed by sphingomyelins (SMs), phosphatidylethanolamines (PEs), phosphatidylserines (PSs), and dihydro-SMs, respectively. A review describing lipid functions in ocular lens has recently been published [21].

The regional distributions of phospholipids in ocular lenses have been assessed using matrix assisted laser desorption ionization time-of-flight (MALDI-TOF) and ^31^P nuclear magnetic resonance (NMR) analyses of extracts of surgically separated concentric sections [22], [23]. In those studies, the concentrations of phospholipids were the highest at the cortex of the lens and decreased toward the lens nucleus. The changes in the distribution of spatial phospholipids were assessed based on the compositional average from relatively large sections of the lens, and therefore the spatial resolution of the experiment was low and no detailed differences in lipid concentrations were observed. The *in situ* spatial analysis of the phospholipids of the lens by direct MALDI-TOF MS analysis was conducted by averaging mass spectra acquired from three or four spots next to each other from the cortex and the nucleus [7]. Although these experiments provided the precise lipid composition at particular spots of the lens, the distribution of lipids in the context of the whole lens remained unclear. As was previously mentioned, several desorption-ionization techniques are available for mass spectrometry imaging and selecting the appropriate technique is crucial. Recently, the research group of Blanksby reported [24] the use of MALDI for mass spectrometric visualization of dihydrosphingomyelin (d18∶0/16∶0) and dihydroceramide (d18∶0/16∶0) in human lenses. Similarly, DESI has served for imaging of cholesterol, sphingomyelins, and glycerophospholipids [25].

We elucidated the spatial distribution of sphingolipids in the porcine lens using MALDI MSI and provided detailed information about compositional changes in the clear lens nucleus and cortex [9]. Following our previous observations, we now provide the whole lipidome of clear porcine lens and also determine the spatial distribution of 30 glycerophospholipids (GPs) using a combination of ultra performance liquid chromatography quadrupole time-of-flight mass spectrometry (UPLC-QTOFMS) and MALDI imaging coupled to Fourier transform ion-cyclotron resonance mass spectrometry (FTICR MS). Although TOF MS is commonly used in MSI, FTICR MS is preferred due to its superior mass resolution and mass accuracy, which allows unambiguous peak recognition and precise assignment of exact masses. Thus, FTICR MS allows for the identification of molecular formulae with increased confidence.

## Results and Discussion

**Figure 1:**
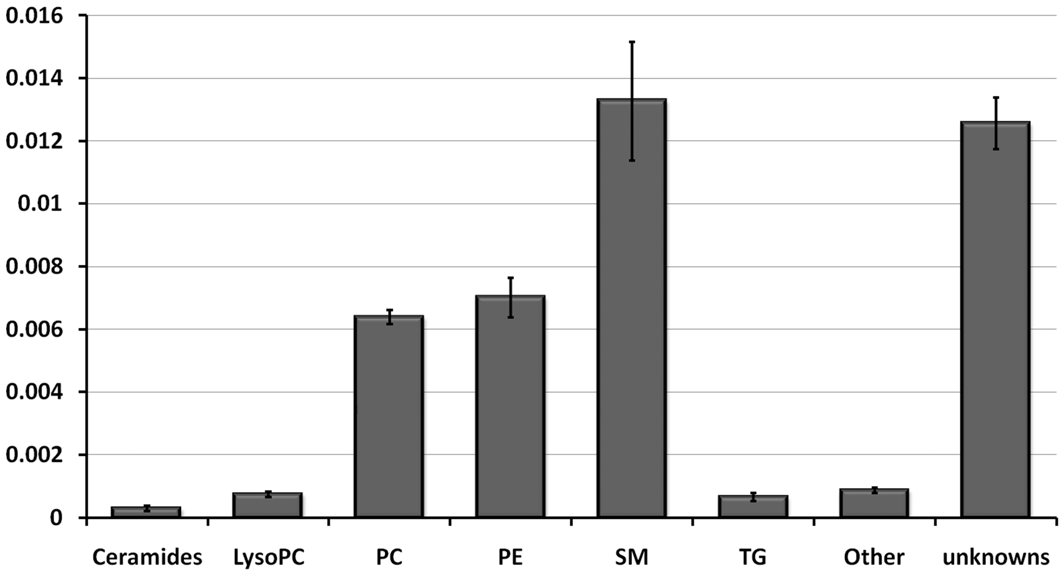
The relative abundance of lipid classes in porcine lens crude lipid extracts based on UPLC-QTOF MS analysis. Each bar represents the mean concentration of six lens extracts. Error bars are standard deviations of six individual measurements. The y-axis is given as nmoles/mg of lens protein. LysoPC, lyso-phosphatidylcholine; PC, phosphatidylcholine; PE, phosphatidylethanolamine; SM, Sphingomyelin; TG, triglyceride.

To begin with, porcine lenses were extracted and analyzed with UPLC-QTOFMS to obtain global lipid profile of the whole lens. The identified lipids are listed in supplementary Table S1, and the most abundant lipid classes are shown in Figure 1. SMs, PCs and PEs were the most abundant identified lipids in the lens. Of these, the number of individual PC species was clearly the highest. The lipid profiles in different lens samples were very similar, the overall deviation of the amount of lipids was on average <30%. These results are in relatively good agreement with Deeley et al. [20]. These authors found that PCs were the most abundant lipids, followed by SMs, PSs, and PEs. Overall, Deeley et al. [20] identified 71 lipid species (22 PEs, 16 PCs, 14 PSs, 13 SMs, 3 phosphatidylinositols, and 3 phosphatidic acids (PAs)). Of these lipids our analysis identified 50%. Most notably due to technical reasons negatively charged phospholipids (such as PSs, phosphatidylinositols, and PAs) were not found among our analysis. However, compared to the study of Deeley and coworkers we were able to identify and quantitate altogether 101 new lipids. Interestingly, 36 triglyceride species were also identified suggesting that some energy storage in epithelial cells has to remain.

**Figure 2:**
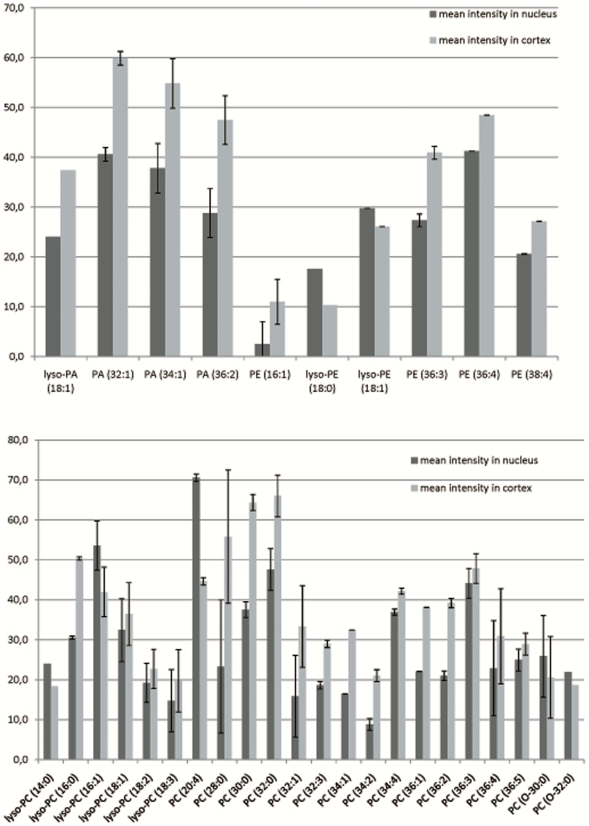
The graphs display mean intensities measured at the cortex (dark gray) and the nucleus (light gray) of the intraocular lens of each lipid. The lipid intensities were measured using matrix-assisted laser desorption ionization mass spectrometry imaging (MALDI MSI) of the lens using ultra-high resolution Fourier transform ion cyclotron resonance mass spectrometry (FTICR MS). Error bars represent standard deviation of 3 samples. Mean intensities can be equally (in relative scale) compared only within one lipid class.

Following our previous study, in which we described the spatial distribution of SMs in porcine lens using MSI [9], we have now resolved the spatial distribution of 30 GPs in the same tissue including 20 PCs, 6 PEs, and 4 PAs (Table 1, Figure 2). It is obvious that a notable fraction of lipid species were not, however, identified in the MSI experiment in comparison to the UPLC-QTOFMS analysis of the whole lens extract. This is partly due to the small sampling area (50 µm) of the MALDI laser in imaging mode. Yet, as can be seen most of the high abundance lipids detected by UPLC-QTOFMS were identified also by MALDI MSI.

**Table 1:** List of identified glyceropospholipids in porcine lens by MALDI MSI.

Compound	Molecular formula	Measured m/z	Error
lyso-PC (14∶0)	C22H46NO7P	490.2905	0.1
lyso-PC (16∶0)	C24H50NO7P	518.3216	−0.2
lyso-PC (16∶1)	C24H48NO7P	516.3059	−0.3
Lyso PC (18∶1)	C26H52NO7P	544.3374	0.0
Lyso PC (18∶2)	C26H50NO7P	542.3233	1.9
Lyso PC (18∶3)	C26H48NO7P	540.3050	−0.7
PC (20∶4)	C28H50NO7P	566.3217	0.0
PC (28∶0)	C36H72NO8P	700.4881	−1.0
PC (30∶0)	C38H76NO8P	728.5199	−0.9
PC (32∶0)	C40H80NO8P	756.5509	−0.6
PC (32∶1)	C40H78NO8P	754.5351	−0.8
PC (32∶3)	C40H74NO8P	752.5192	−0.8
PC (34∶1)	C42H82NO8P	782.5661	−0.5
PC (34∶2)	C42H80NO8P	780.5510	−0.5
PC (34∶4)	C42H76NO8P	778.5353	1.4
PC (36∶1)	C44H86NO8P	810.5983	0.0
PC (36∶2)	C44H84NO8P	808.5832	0.6
PC (36∶3)	C44H82NO8P	806.5664	−0.7
PC (36∶4)	C44H80NO8P	804.5507	−0.7
PC (36∶5)	C44H78NO8P	802.5367	1.4
lyso-PE (16∶1)	C21H42NO7P	474.2595	−1.6
lyso-PE (18∶0)	C23H48NO7P	504.3075	1.8
lyso-PE (18∶1)	C23H46NO7P	502.2901	−0.6
PE (36∶3)	C41H76NO8P	764.5204	0.7
PE (36∶4)	C41H74NO8P	762.5054	1.2
PE (38∶4)	C43H78NO8P	790.5355	0.1
lyso-PA (18∶1)	C21H41O7P	459.2481	−0.3
PA (32∶1)	C35H67O8P	669.4462	−0.6
PA (34∶1)	C37H71O8P	697.4772	1.0
PA (36∶2)	C39H73O8P	723.4928	1.0

Identification of all compounds was based on their exact mass assignment of sodium adducts (measured m/z). Molecular formula shows elemental composition of the uncharged molecule.

**Figure 3:**
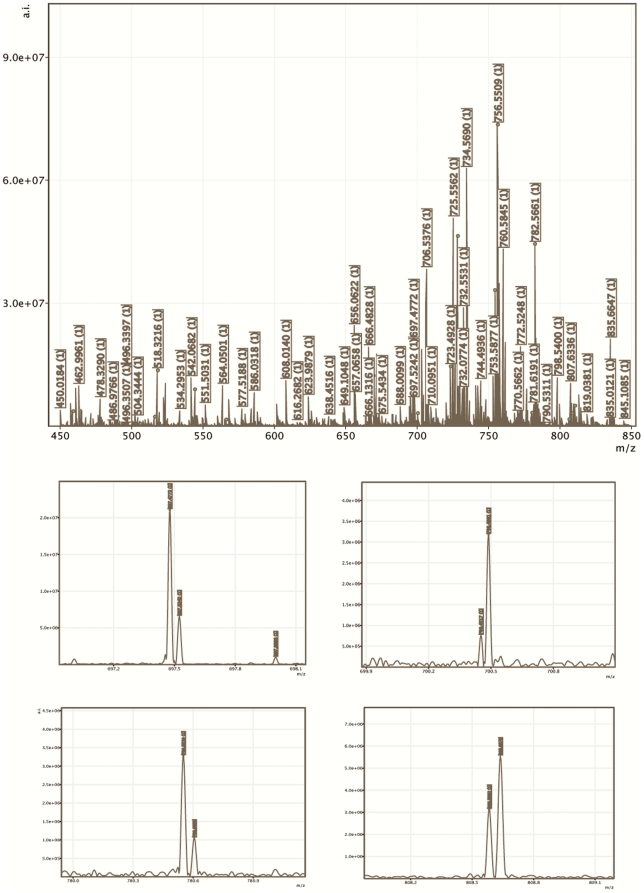
Fourier transform ion cyclotron resonance mass spectrometry (FTICR MS) mass spectra displayed in the mass range of identified lipids (top) and four selected zoomed spectra (bottom). In the top spectra, circle on a crest of mass peaks indicates sodium adducts of the identified glyceroposholipids. At the bottom, we demonstrate using nominal *m/z* 697, 700, 780, and 808 the importance of the use of high-resolution MS in imaging. Analyte peaks are separated from interfering biological and MALDI matrix peaks.

Figure 3 displays averaged mass spectra in the mass range of all identified lipid species and also several zoomed parts. The enlargements demonstrate how important it is to use ultra high-resolution mass spectrometry for imaging. Analyte peaks are separated from peaks originating from the biological and MALDI matrices. The use of lower resolution instrumentation would result in overlapping of analyte peaks with the interferences. As a consequence, the spatial distribution of lipids can be compromised. In our experiment we observed interferences in proximity of glycerophosholipid peaks (shown only for sodium adducts) at the following nominal masses: 506, 697, 700, 723, 728, 754, 780, 806, 808, and 810 m/z.

PCs composed the largest group by number of lipids identified by MALDI MSI in keeping with UPLC-QTOFMS and also in line with findings from other epithelia cells, in which PCs are the major lipid class. For PCs with 14–20 (lyso-forms) and 34–36 carbons in their acyl chains, the mean intensity of the lipids was higher in the cortex than in the nucleus. These findings are in accordance with the previous work of Rujoi et al. [7]. In contrast, PCs with 20–32 carbons in their acyl chains had higher mean lipid intensities in the nucleus compared to the cortex. Markedly these lipids were saturated in contrast to all other diacyl lipids identified. The high concentration of saturated and long acyl chain lipids make the cellular membranes significantly more rigid in the nucleus compared to the fluid like membranes in the cortex. Also the higher concentration of rigid SMs [9] in the nucleus further rigidifies the plasma membrane. This finding may reflect the slow metabolism of the nuclear fiber cells. PCs were identified as protonated, sodium, and potassium adducts. It should be noted that comparison of mean intensities is only relevant within one lipid class (Figure 2), since ionization desorption efficacy differs from one lipid class to another.

The second most abundant group of lipids consisted of 6 PEs∶ lysoPE (16∶1), lysoPE (18∶0), and lysoPE (18∶1), which all were identified as sodium and potassium adducts, had a higher mean intensity in the cortex than in the nucleus. In contrast, PEs containing two acyl chains (PE(36∶3), PE(36∶4), and PE(38∶4)) had a slightly higher abundance in the nucleus compared to the cortex. The distribution of diacyl and unsaturated PEs was just the opposite compared to diacyl unsaturated PCs (Fig. 2).

Finally, 4 PAs were also identified and their spatial distribution resolved: a single lysoPA (18∶1), and diacyl PA (32∶1), PA (34∶1), and PA (36∶2). All lipids were identified as sodium and potassium adducts and two of these PAs ((PA (34∶1) and PA (36∶2)) were among those detected also by Deeley et al. [20]. All PAs had a higher mean intensity in the nucleus compared to the cortex.

**Figure 4:**
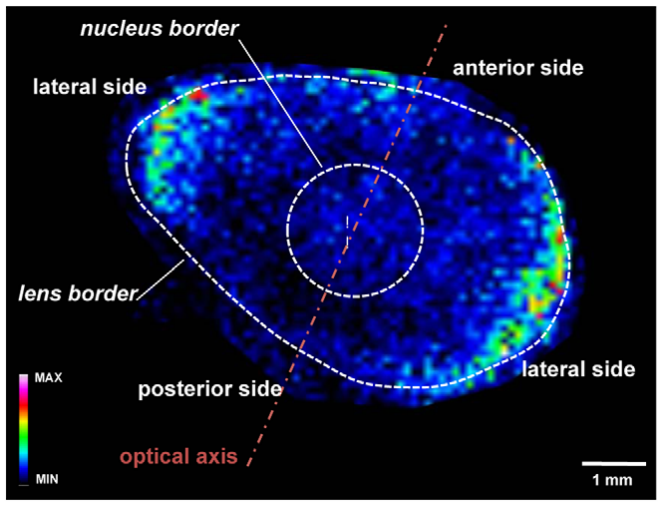
Molecular image of PC (34∶2) used to visualize structures in an ocular lens using matrix-assisted laser desorption ionization mass spectrometry imaging (MALDI MSI). The lens cortex is the area between the nucleus and lens borders. Equatorial margins are clearly defined by the high abundance of phospholipids, and the lens nucleus is less visible in this case due to the changes in lipid composition in the lens.

Figure 4 presents a typical image of the spatial distribution of PC (34∶2). The nucleus and the cortex are easily visualized through the changes in the spatial concentration of this PC as well as the fine structures of the lens, since the PC localizes to the equatorial margins where the germination zone resides. In the adult lens, almost none of the epithelial fiber cells are dividing. In the germinal zone, the lens epithelial cells proliferate slowly. This suggests that the newly formed cells contain high amounts of PCs with relatively high amounts of intracellular organelles. As the cells differentiate and the intracellular organelles are lost [2], the relative content of PC decreases.

**Figure 5:**
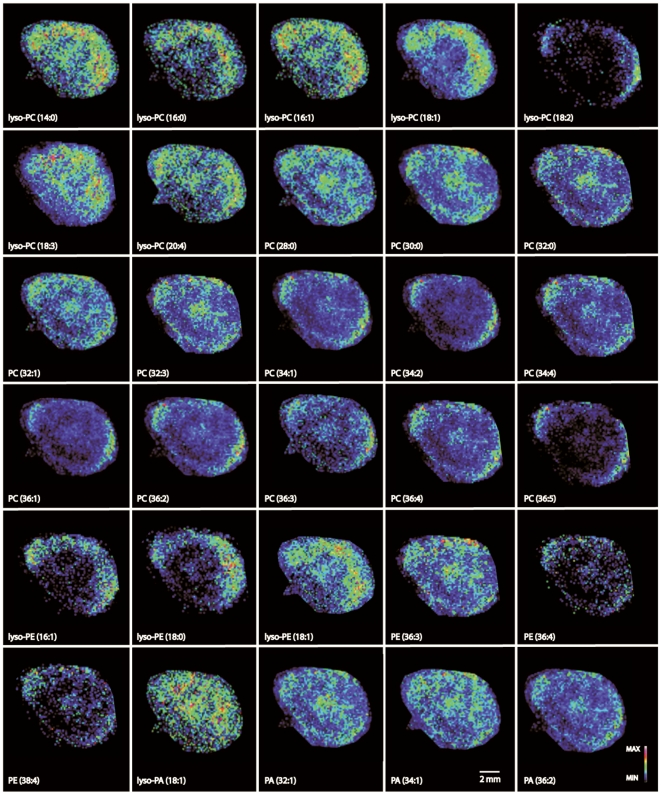
Spatial distribution of glycerophosphocholines (PCs), glycerophosphatidylethanolamine (PE) and glycerophosphates (PAs) visualized in false colors. MALDI-MS images were acquired by an APEX Ultra 9.4 T FT-ICR mass spectrometer equipped with a Smart beam laser. The molecular images of the lipids were visualized using the FlexImaging software, and the intensity of the image was normalized to the total ion chromatogram. Identification of lipids was performed from the average spectra of the entire lens using Mass data analysis software.

A trend in spatial distribution as a function of acyl chain length can be observed for all GPs analyzed (Figure 5). LysoGPs, except lysoPA, exhibit higher mean intensities in the cortex than in the nucleus (Figures 2 & 5), and they are distributed evenly across the whole cortex area. On the contrary, GPs with two saturated acyl chains have a higher mean intensity in the nucleus than in the cortex (except for PCs with acyl chains of 18 carbons, which have the same mean intensity in the nucleus and the cortex) and tend to localize to the equatorial margins. It is presently unknown how this concentration gradient is maintained. Degradation would decrease the lysolipid content toward the nucleus. It is quite unlikely that the lysolipids would be acylated as the lens matures. Alternatively, in the developing lens high amounts of lysolipids could act as surfactants and hamper the development of the lens cortex, whereas the mature lens may accommodate the lysolipids more easily.

The spatial distribution of lipids described here correlates well with previous observations on the spatial distribution of the most abundant integral lens membrane protein [27]. Full-length AQP0 was found throughout the lens, but its concentration increased toward the periphery of the lens. In contrast, the major truncation product of AQP0 (amino acids 1–246) was absent from the lens periphery but was present and increased in abundance closer to the nucleus. In keeping with previous ESI-MS experiment [20] our results suggest that SMs and PCs are the most abundant lipids in the porcine lens. PCs are almost universally the most abundant lipid species across different species as well as in various cell types and are believed to provide an optimal platform for the functioning of integral and soluble membrane proteins. In many cell types, PCs are also important sources of lipid second messengers, but in metabolically inactive tissues such as the lens, this property/function seems unlikely. The presence of saturated acyl chains increases toward the nucleus (see PC (28∶0) and PC (30∶0)). As we have previously shown, this trend occurs with all but one identified SM [9] and suggests that these lipids play a functional role in the lens nucleus by providing stiffer membranes that are more resistant to various stress factors. In contrast, the polyunsaturated PCs are found primarily in the cortical regions of the lens. PAs and PEs, which have a major effect on the lateral pressure profile of membranes [26], show a similar spatial distribution, whereas the relative concentrations of PAs and PEs are just the opposite; the concentration of PAs increases toward the nucleus, whereas PEs are found at higher concentrations in the cortex. These diffzerences may play a role in the regulation of whole length AQP0 and the truncation product of AQP0.

In conclusion, UPLC-QTOFMS unambiguously identified lipids from tissue samples through the determination of their molecular formulae by exact mass measurements. By FTICR ultra-high resolution mass spectrometry we were now able to provide the spatial lipid distribution of >20% of all lipids in the porcine lens. Since tandem mass spectrometry of lipids ionized directly from the tissue sample is not feasible for most of the identified lipids due to their low ion intensities, this method represents an elegant and reliable analytical approach. The spatial distribution of GPs provided by mass spectrometry imaging reveals the organization of lipid membranes in a healthy porcine ocular lens. These data along with the known spatial organization of membrane proteins supplements current knowledge of cell membrane functions. The long-term goal of our ongoing studies is to characterize the lipid distribution of clear and cataractous human lenses and thus provide mechanisms to understand diseases of the lens.

## Materials and Methods

### Basic Experimental Principle

We used MALDI MSI to investigate the spatial distribution of GPs in a porcine ocular lens. In MALDI MSI, compounds are ionized directly from the tissue slice, which is attached on a conductive surface moving in set raster steps under a laser beam. Mass spectra collected from each point within the raster are used to assemble the molecular images in correlation with their *x-* and *y-* positions. The tissue surface is uniformly coated by the deposition of a matrix aerosol to ensure co-crystallization with lipids, which facilitates desorption and ionization. The coating process is computer controlled to avoid excessive over-wetting, which would deteriorate the spatial distribution of the molecules in the tissue. Ions desorbed from the tissue are accelerated into the mass spectrometer for separation based on the mass-to-charge ratios of the ions and detected. Sampling of the ions directly from the tissue into the mass spectrometer provides very rich mass spectra with respect to the number of ions, so a high-resolution mass detector is needed for exact identification of the ions.

### Tissue Preparation

All experiments were performed in accordance with the guidelines of the Public Health Service Policy on Humane Care and Use of Laboratory Animals and the Ethical Committees of all institutions that have approved this research. Porcine eyes from 2 year old animals were provided by a local abattoir (LSO Foods Oy, Lahti, Finland) and transported to the laboratory within 6 h of sacrifice. The ocular lenses were immediately excised and stored in a physiological saline solution at 4°C for transportation to the MS laboratory. The tissues were frozen over liquid nitrogen and stored at −80°C.

For the MALDI analyses, the tissues were cut in a microcryotome (Leica CM 1950, Leica Biosystem, Germany) along the equatorial plane to generate 12 µm slices. The slices were then transferred and attached onto an ITO glass slide coated with a thin layer of ethanol (2 µL). The lens structure is extremely fragile when cut with a microcryotome into thin slices, so ethanol [27] or methanol [28], [29] soft-landing procedures for the tissue on the MALDI surface have been used to fix the tissue structure for MALDI MSI analysis of proteins. Our previous work on sphingolipids has demonstrated that the use of ethanol does not affect the spatial distribution of lipids [9]. After drying the samples for 10 min in a desiccator, the tissue was coated with α-cyano-4-hydroxycinnamic acid (CHCA) MALDI matrix using an ImagePrep (Bruker Daltonics, Germany) device. CHCA was prepared in 50/50% (v/v) acetonitrile/trifluoroacetic acid (0.2%) at a concentration of 7 mg/mL. A Nikon scanner (Coolscan VED, Nikon, Tokyo, Japan) was used for optical scanning of the tissues. The samples were analyzed immediately after MALDI matrix deposition.

For the UPLC-QTOFMS analyses, a standard mixture 1 (10 µl) containing PC(17∶0/0∶0), PC(17∶0/17∶0), PE(17∶0/17∶0) and Cer(d18∶1/17∶0), (Avanti Polar Lipids, Inc.) and TG(17∶0/17∶0/17∶0) (Larodan Fine Chemicals) was added to the lens samples before homogenization The samples were homogonised, with the HPLC-grade chloroform and methanol (3∶1; 3000 µL). The homogenate was mixed with 1.5 ml of 0.15 M NaCl (0.9%), vortexed for 1 min, and allowed to stand for 30 min. Subsequently, samples were centirfuged and the lower phase was collected and internal standard mixture 2 is added. The internal standard mixture 2 contained the labeled lipids PC (16∶1/0∶0-D_3_), PC(16∶1/16∶1-D_6_) and TG(16∶0/16∶0/16∶0-^13^C3).

### MALDI-MS Imaging

MALDI-MS images were acquired by an APEX Ultra 9.4 T FT-ICR (dual ion source ESI/MALDI, Bruker Daltonics, Germany) mass spectrometer equipped with a Smart beam laser (200 Hz, spot diameter 50 µm). The entire area of the porcine lens was examined with the laser with a raster step size of 150 µm. The laser performed 600 shots per spot and single spectra were collected from one spot. The mass spectra were acquired in positive ion mode and the mass range was 200–1100 m/z. The mass resolution at 400 m/z was 66,000. The time fill for the ICR cell was 1400 µs. After analysis, the spectra were apodized using square sin apodization with one zero fill. Analysis of the entire tissue lasted about 8 h, and the resulting dataset was approximately 10 GB in size. The molecular images of the lipids were visualized using the FlexImaging software (version 2.1, Bruker Daltonics, Germany), and the intensity of the image was normalized to the total ion chromatogram. Identification of lipids was performed from the average spectra of the entire lens using Mass data analysis software [30]. First, the spectra were internally calibrated against the five selected cluster ions of the CHCA MALDI matrix, and the mass peaks were deisotoped and then searched within an internal database adopted from the LIPID MAPS database [31]. The tolerance for the search was 2 ppm, and protonated ions and sodium and potassium adducts were included. The local intensity of a lipid species was determined by a local Mean Gray Value at a grayscale molecular image in ImageJ image processing software (National Institutes of Health, USA, rsbweb.nih.gov/ij/) using the function Measure. The relative abundance of a lipid was calculated from the mass peak intensities of all identified lipids in the averaged mass spectra. In cases where a lipid was presented in multiple ion forms (e.g., as a protonated ion and/or sodium and potassium adducts), their intensities were summed. The lipid abundances were normalized to the most abundant peak in the mass spectra. Note that different lipid classes have different responses in MALDI, and it is unfortunately not feasible to quantify lipids directly from a tissue. Nevertheless, the measured intensities were in accordance with previously reported values [7], [32].

### Lipid profiling by UPLC-QTOFMS

The lens lipid extracts were analyzed on a Waters Q-Tof Premier mass spectrometer combined with an Acquity Ultra Performance LC™ (UPLC). The column (at 50°C) was an Acquity UPLC™ BEH C18 2.1×100 mm with 1.7 µm particles. The solvent system included (A) ultrapure water (1% 1 M NH_4_Ac, 0.1% HCOOH) and (B) LC/MS grade acetonitrile/isopropanol (1∶1, 1% 1 M NH_4_Ac, 0.1% HCOOH). The gradient started from 65% A/35% B, reached 80% B in 2 min, 100% B in 7 min, and remained there for 7 min. The flow rate was 0.400 ml/min and the injected amount was 2.0 µl (Acquity Sample Organizer, at 10°C). Reserpine was used as the lock spray reference compound. The lipid profiling was carried out using ESI in positive mode and the data was collected at a mass range of m/z 300–1200 with scan duration of 0.2 sec.

The data processing using MZmine2 software included alignment of peaks, peak integration, normalization, and peak identification. Lipids were identified using an internal spectral library. The data was normalized using one or more internal standards representatives of each class of lipid present in the samples: the intensity of each identified lipid was normalized by dividing it with the intensity of its corresponding standard and multiplying it by the concentration of the standard. All monoacyl lipids except cholesterol esters, such as monoacylglycerols and monoacylglycerophospholipids, were normalized with PC(17∶0/0∶0), all diacyl lipids except ethanolamine phospholipids were normalized with PC(17∶0/17∶0), all ceramides with Cer(d18∶1/17∶0), all diacyl ethanolamine phospholipids with PE(17∶0/17∶0), and TG and cholesterol esters were normalized with TG(17∶0/17∶0/17∶0). Other (unidentified) molecular species were normalized with PC(17∶0/0∶0) for retention time <300 s, PC(17∶0/17∶0) for retention time between 300 s and 410 s, and TG(17∶0/17∶0/17∶0) for higher retention times. Finally, the lipids were further normalized by dividing the normalized lipid concentration with the protein content of the sample.

## Supporting Information

Table S1 Lipids identified with UPLC-QTOFMS. Identification was based on in-house library, with maximum allowed difference of retention times 7 s and m/z value 0.04.
(DOC)
