# Supplementary material for: Spatial Distribution of Glycerophospholipids in the Ocular Lens
Source: PLoS One. 2011 Apr 29;6(4):e19441. doi: 10.1371/journal.pone.0019441 (PMC3084859; doi:10.1371/journal.pone.0019441)
Supplement: Table S1 — Lipids identified with UPLC-QTOFMS. Identification was based on in-house library, with maximum allowed difference of retention times 7 s and m/z value 0.04. (DOC) [file pone.0019441.s001.doc]

| **m/z** | **Retention time (s)** | **Name** | **nmol/mg** |
| --- | --- | --- | --- |
| 568.56778 | 411.8 | Cer(d18:0/18:0) | 0.010828 |
| 602.58805 | 417.6 | Cer(d18:0/23:0) | 0.039033 |
| 616.60036 | 430.2 | Cer(d18:0/24:0) | 0.011237 |
| 520.50827 | 347.4 | Cer(d18:1/16:0) | 0.01195 |
| 548.54119 | 397.3 | Cer(d18:1/18:0) | 0.01319 |
| 562.54337 | 410.1 | Cer(d18:1/19:0) | 0.00398 |
| 576.56367 | 420.3 | Cer(d18:1/20:0) | 0.007142 |
| 622.61585 | 432.5 | Cer(d18:1/22:0) | 0.005365 |
| 604.57127 | 436.3 | Cer(d18:1/22:0) | 0.015575 |
| 632.62964 | 434.2 | Cer(d18:1/24:0) | 0.107151 |
| 630.60670 | 431.4 | Cer(d18:1/24:1) | 0.058991 |
| 468.31089 | 153.5 | LysoPC(14:0) | 0.016532 |
| 496.34085 | 175.6 | LysoPC(16:0) | 0.458578 |
| 494.32526 | 159.0 | LysoPC(16:1) | 0.01548 |
| 524.37169 | 199.9 | LysoPC(18:0) | 0.050146 |
| 508.37712 | 208.6 | LysoPC(18:1e) | 0.021281 |
| 520.33741 | 166.7 | LysoPC(18:2) | 0.003015 |
| 518.32058 | 177.2 | LysoPC(18:3) | 0.039185 |
| 504.33623 | 183.2 | LysoPC(18:3e)+LysoPE(20:3) | 0.002726 |
| 502.32113 | 182.9 | LysoPC(18:4e)+LysoPE(20:4) | 0.004482 |
| 546.35749 | 170.8 | LysoPC(20:3) | 0.00801 |
| 532.33820 | 187.5 | LysoPC(20:3e) | 0.105341 |
| 544.34179 | 158.3 | LysoPC(20:4) | 0.005278 |
| 544.33815 | 178.6 | LysoPC(20:4) | 0.01856 |
| 452.31603 | 160.9 | LysoPE(16:1) | 0.007293 |
| 482.32655 | 164.9 | LysoPE(18:0) | 0.010808 |
| 478.33131 | 175.7 | LysoPE(18:2) | 0.013502 |
| 490.29114 | 153.7 | LysoPE(19:3) | 0.002335 |
| 508.37001 | 188.0 | LysoPE(20:1) | 0.010788 |
| 504.34097 | 178.3 | LysoPE(20:3) | 0.002426 |
| 561.52285 | 364.9 | PA(33:2) | 0.002996 |
| 678.50728 | 304.4 | PC(28:0) | 0.404954 |
| 706.53899 | 337.5 | PC(30:0) | 1.052475 |
| 690.53899 | 351.3 | PC(30:1e) | 0.115436 |
| 718.57637 | 357.6 | PC(31:1)+PE(34:1) | 0.100465 |
| 718.56877 | 381.1 | PC(31:1)+PE(34:1) | 0.18452 |
| 734.56950 | 368.7 | PC(32:0) | 1.161326 |
| 732.55368 | 338.1 | PC(32:1) | 0.492763 |
| 716.55953 | 402.2 | PC(32:2e) | 0.026004 |
| 728.52126 | 334.7 | PC(32:3) | 0.082824 |
| 700.52516 | 361.1 | PC(32:3e) | 0.070916 |
| 724.51177 | 331.8 | PC(32:5) | 0.016775 |
| 724.51608 | 356.3 | PC(32:5) | 0.067075 |
| 744.58822 | 358.7 | PC(33:2)+PE(36:2) | 0.008191 |
| 744.58105 | 379.8 | PC(33:2)+PE(36:2) | 0.188682 |
| 742.56268 | 399.8 | PC(33:3)+PE(36:3) | 0.008267 |
| 762.60395 | 395.1 | PC(34:0) | 0.337157 |
| 748.61858 | 411.3 | PC(34:0e) | 0.009599 |
| 760.58699 | 365.2 | PC(34:1) | 0.859694 |
| 746.58229 | 401.8 | PC(34:1e)+PE(37:1e) | 0.063922 |
| 746.60536 | 384.0 | PC(34:1e)+PE(37:1e) | 0.13033 |
| 758.57182 | 346.6 | PC(34:2) | 0.099358 |
| 756.55342 | 320.8 | PC(34:3) | 0.033209 |
| 754.53821 | 334.5 | PC(34:4) | 0.046146 |
| 752.51359 | 329.6 | PC(34:5) | 0.014073 |
| 774.60153 | 380.3 | PC(35:1) | 0.04997 |
| 788.62306 | 392.7 | PC(36:1) | 0.196629 |
| 786.59980 | 364.4 | PC(36:2) | 0.083889 |
| 784.58489 | 357.8 | PC(36:3) | 0.068522 |
| 782.56937 | 333.6 | PC(36:4) | 0.118219 |
| 780.58466 | 395.1 | PC(36:5) | 0.033742 |
| 778.53681 | 330.0 | PC(36:6) | 0.009076 |
| 800.61894 | 382.1 | PC(37:2) | 0.002435 |
| 796.59966 | 383.6 | PC(37:4)/PE(40:4) | 0.023328 |
| 794.59575 | 373.9 | PC(37:5)+PE(40:5) | 0.001288 |
| 816.68597 | 416.1 | PC(38:1) | 0.04635 |
| 802.66969 | 414.5 | PC(38:1e) | 0.004409 |
| 814.63407 | 394.7 | PC(38:2) | 0.019244 |
| 812.61613 | 377.4 | PC(38:3) | 0.033331 |
| 810.60058 | 357.4 | PC(38:4) | 0.035996 |
| 794.59977 | 352.4 | PC(38:5e) | 0.005388 |
| 806.56939 | 322.1 | PC(38:6) | 0.005816 |
| 804.55061 | 333.6 | PC(38:7) | 0.016979 |
| 790.59702 | 418.2 | PC(38:7e) | 0.0195 |
| 842.65974 | 415.7 | PC(40:2) | 0.01195 |
| 840.65292 | 401.0 | PC(40:3) | 0.003127 |
| 838.63326 | 376.2 | PC(40:4) | 0.006881 |
| 824.62649 | 406.1 | PC(40:4e) | 0.00457 |
| 822.62266 | 385.6 | PC(40:5e) | 0.003184 |
| 834.59967 | 347.0 | PC(40:6) | 0.001611 |
| 830.56980 | 332.8 | PC(40:8) | 0.008371 |
| 702.54299 | 390.6 | PE(16:0p/18:1) | 1.854119 |
| 728.55691 | 387.4 | PE(18:0p/18:2)+PE(18:1p/18:1) | 3.016704 |
| 660.49404 | 346.7 | PE(30:2) | 0.001501 |
| 692.55723 | 357.4 | PE(32:0) | 0.164559 |
| 690.54036 | 381.0 | PE(32:1) | 0.003822 |
| 686.52241 | 346.8 | PE(32:3) | 0.001309 |
| 672.49004 | 350.5 | PE(32:3e) | 0.001005 |
| 704.55939 | 393.4 | PE(34:1e) | 0.043173 |
| 716.54956 | 351.1 | PE(34:2) | 0.070892 |
| 688.52603 | 375.8 | PE(34:2e) | 0.017765 |
| 712.51506 | 364.9 | PE(34:4) | 0.001573 |
| 698.50695 | 350.0 | PE(34:4e) | 0.016 |
| 746.56954 | 352.4 | PE(36:1) | 0.134425 |
| 730.57512 | 413.7 | PE(36:2e) | 0.201446 |
| 742.55013 | 351.0 | PE(36:3) | 0.013493 |
| 714.53950 | 375.1 | PE(36:3e) | 0.018468 |
| 740.53276 | 381.2 | PE(36:4) | 0.002212 |
| 726.55376 | 345.5 | PE(36:4e) | 0.0018 |
| 726.53130 | 379.1 | PE(36:4e) | 0.046875 |
| 710.54804 | 387.0 | PE(36:5e) | 0.001217 |
| 724.51775 | 392.0 | PE(36:5e) | 0.003293 |
| 776.61775 | 407.1 | PE(38:0) | 0.004979 |
| 772.60160 | 404.7 | PE(38:2) | 0.029016 |
| 770.58910 | 378.8 | PE(38:3)+PC(35:3) | 0.079554 |
| 768.56542 | 362.7 | PE(38:4) | 0.030742 |
| 768.55796 | 372.0 | PE(38:4)+PC(35:4) | 0.003255 |
| 754.56520 | 404.2 | PE(38:4e) | 0.023269 |
| 766.54910 | 347.1 | PE(38:5) | 0.015802 |
| 753.55149 | 360.1 | PE(38:5e) | 0.006508 |
| 750.53998 | 355.0 | PE(38:6e) | 0.154823 |
| 762.53432 | 353.0 | PE(38:7) | 0.025461 |
| 748.52827 | 345.2 | PE(38:7e) | 0.003746 |
| 798.60518 | 408.0 | PE(40:3) | 0.014457 |
| 782.59997 | 411.4 | PE(40:4e) | 0.01202 |
| 792.57528 | 346.8 | PE(40:6) | 0.016418 |
| 778.54731 | 410.4 | PE(40:6e) | 0.001074 |
| 790.55497 | 382.1 | PE(40:7) | 0.012028 |
| 776.55030 | 363.1 | PE(40:7e) | 0.00427 |
| 788.54027 | 349.6 | PE(40:8) | 0.017854 |
| 774.54344 | 344.1 | PE(40:8e) | 0.006095 |
| 700.52462 | 368.6 | PE(P-16:0/18:2) | 0.134396 |
| 752.55268 | 377.8 | PE(P-18:0/20:4)+PE(P-16:0/22:4) | 0.734399 |
| 778.56805 | 373.8 | PE(P-18:0/22:5) | 0.063647 |
| 675.54432 | 307.5 | SM(d18:1/14:0) | 0.337294 |
| 701.55851 | 310.5 | SM(d18:1/16:1) | 0.035715 |
| 731.60522 | 373.3 | SM(d18:1/18:0) | 2.933128 |
| 787.66918 | 407.4 | SM(d18:1/22:0) | 0.796266 |
| 801.68694 | 439.2 | SM(d18:1/23:0) | 0.519223 |
| 815.70169 | 432.1 | SM(d18:1/24:0) | 0.143358 |
| 813.68457 | 421.2 | SM(d18:1/24:1) | 8.505054 |
| 824.77551 | 536.4 | TG(14:0/16:0/18:0) | 0.007186 |
| 848.77179 | 510.1 | TG(14:0/18:1/18:1)+TG(16:0/16:1/18:1) | 0.016709 |
| 866.82092 | 573.4 | TG(16:0/18:0/17:0) | 0.301398 |
| 878.82156 | 554.2 | TG(16:0/18:0/18:1) | 0.005922 |
| 876.80322 | 527.6 | TG(16:0/18:1/18:1) | 0.019232 |
| 874.78888 | 508.2 | TG(16:0/18:2/18:1) | 0.063072 |
| 872.77440 | 493.1 | TG(18:1/16:1/18:2)+TG(18:2/18:2/16:0) | 0.003418 |
| 740.68186 | 482.6 | TG(42:0) | 0.000798 |
| 768.70893 | 497.7 | TG(44:0) | 0.001599 |
| 766.69955 | 481.0 | TG(44:1) | 0.000613 |
| 758.60466 | 415.0 | TG(44:5) | 0.005753 |
| 756.58801 | 410.2 | TG(44:6) | 0.057631 |
| 794.72742 | 495.7 | TG(46:1) | 0.004529 |
| 794.69626 | 447.4 | TG(46:1)* | 0.001146 |
| 792.71274 | 480.2 | TG(46:2) | 0.001084 |
| 784.61728 | 417.3 | TG(46:6) | 0.003305 |
| 808.74264 | 503.9 | TG(47:1)* | 0.001079 |
| 800.64911 | 419.1 | TG(47:5) | 0.053771 |
| 820.74331 | 494.9 | TG(48:2) | 0.006898 |
| 818.72222 | 475.6 | TG(48:3) | 0.001245 |
| 834.76194 | 502.0 | TG(49:2)* | 0.00111 |
| 832.73982 | 537.3 | TG(49:3) | 0.02357 |
| 828.64557 | 404.7 | TG(49:5) | 0.001002 |
| 852.80661 | 559.9 | TG(50:0) | 0.002494 |
| 850.78082 | 532.0 | TG(50:1) | 0.01645 |
| 846.75781 | 493.5 | TG(50:3) | 0.005372 |
| 844.67881 | 437.2 | TG(50:4) | 0.017156 |
| 860.77785 | 500.6 | TG(51:3) | 0.00065 |
| 880.83874 | 588.0 | TG(52:0) | 0.001151 |
| 872.71382 | 456.3 | TG(52:4) | 0.0069 |
| 870.69576 | 435.1 | TG(52:5) | 0.012099 |
| 866.71975 | 470.9 | TG(52:7) | 0.000722 |
| 894.85459 | 602.9 | TG(53:0) | 0.000827 |
| 890.81751 | 538.4 | TG(53:2) | 0.000865 |
| 888.81293 | 516.7 | TG(53:3) | 0.000732 |
| 882.73664 | 455.6 | TG(53:6) | 0.003871 |
